# Fabrication of Hydrogen Boride Thin Film by Ion Exchange in MgB_2_

**DOI:** 10.3390/molecules26206212

**Published:** 2021-10-14

**Authors:** T. Hirabayashi, S. Yasuhara, S. Shoji, A. Yamaguchi, H. Abe, S. Ueda, H. Zhu, T. Kondo, M. Miyauchi

**Affiliations:** 1 Department of Materials Science and Engineering, School of Materials and Chemical Technology, Tokyo Institute of Technology, Tokyo 152-8552, Japan; hirabayashi.t.ac@m.titech.ac.jp (T.H.); yasuhara.s.aa@m.titech.ac.jp (S.Y.); shoji.s.chem@gmail.com (S.S.); ayamaguchi@ceram.titech.ac.jp (A.Y.); 2 Department of Mechanical Engineering, School of Materials Science and Engineering, Tsinghua University, Beijing 100084, China; hongweizhu@tsinghua.edu.cn; 3 Department of Materials Science & Engineering, Cornell University, Ithaca, NY 14853, USA; 4 Center for Green Research on Energy and Environmental Materials, National Institute for Materials Science, Tsukuba 305-0044, Japan; ABE.Hideki@nims.go.jp; 5 Synchrotron X-ray Station at SPring-8, National Institute for Materials Science, Hyogo 679-5148, Japan; UEDA.Shigenori@nims.go.jp; 6 Research Center for Advanced Measurement and Characterization, National Institute for Materials Science, Tsukuba 305-0047, Japan; 7 Research Center for Functional Materials, National Institute for Materials Science, Tsukuba 305-0044, Japan; 8 Department of Materials Science and Tsukuba Research Center for Energy Materials Science, Faculty of Pure and Applied Sciences, University of Tsukuba, Tsukuba 305-8573, Japan; 9 Materials Research Center for Element Strategy, Tokyo Institute of Technology, Yokohama 226-8503, Japan

**Keywords:** hydrogen boride, thin film, ion-exchange, hydrogen release, pulsed laser deposition

## Abstract

In this study, hydrogen boride films are fabricated by ion-exchange treatment on magnesium diboride (MgB_2_) films under ambient temperature and pressure. We prepared oriented MgB_2_ films on strontium titanate (SrTiO_3_) substrates using pulsed laser deposition (PLD). Subsequently, these films were treated with ion exchangers in acetonitrile solution. TOF-SIMS analysis evidenced that hydrogen species were introduced into the MgB_2_ films by using two types of ion exchangers: proton exchange resin and formic acid. According to the HAXPES analysis, negatively charged boron species were preserved in the films after the ion-exchange treatment. In addition, the FT-IR analysis suggested that B-H bonds were formed in the MgB_2_ films following the ion-exchange treatment. The ion-exchange treatment using formic acid was more efficient compared to the resin treatment; with respect to the amount of hydrogen species introduced into the MgB_2_ films. These ion-exchanged films exhibited photoinduced hydrogen release as observed in a powder sample. Based on the present study, we expect to be able to control the morphology and hydrogen content of hydrogen boride thin films by optimising the ion-exchange treatment process, which will be useful for further studies and device applications.

## 1. Introduction

In contrast to their bulk counterparts, two-dimensional materials, such as graphene, exhibit unique electronic properties and ultra-high specific surface areas. These unusual characteristics result in energy band structures sensitive to external perturbations and attract significant research interest [1]. Recently, Kondo et al. successfully synthesised hydrogen boride (HB) nanosheets [2]. The synthesis method involves the 3-day reaction of layered bulk magnesium diboride (MgB_2_) powder with an ion-exchange resin in an organic solvent to promote the exfoliation of MgB_2_. This is the first experimentally synthesised borophane (hydrogenated borophene), which is composed of sp^2^-bonded boron, forming hexagonal boron networks with bridge hydrogens. The chemical composition of the material is HB, where the hydrogen/boron atomic ratio is 1/1. Previous experiments have shown that hydrogen boride sheets have a high H_2_ content (8.5 wt %) [2], unique electronic properties [3,4], solid acid catalytic properties [5,6], photoinduced hydrogen release capability [7], and a unique ability to reduce metal ions to form nanocomposites [8,9,10]. In addition, theoretical models and computer simulations demonstrate that borophane is a promising candidate for applications such as anode material, alcohol gas sensors, current limiters, and photodetectors [11,12,13,14].

At present, studies on the synthesis of hydrogen boride have been carried out only for a powder sample obtained from finely dispersed MgB_2_ [2,3,4,5,6,7,8,9,10]. However, hydrogen boride thin films deposited on substrates would offer better opportunities for the in-depth analysis of the physical properties and investigation of possible applications of this material. Thus far, there has been no research on well-defined thin films of hydrogen boride. The epitaxial growth of hydrides is quite a challenging objective, owing to their thermal and chemical instability in air and multiple charged states [15,16,17].

Herein, we have developed a facile method for the fabrication of thin films of hydrogen boride using an ion-exchange treatment of deposited MgB_2_ films under ambient temperature and pressure. Firstly, oriented MgB_2_ films on SrTiO_3_ (STO) substrates were prepared by pulsed laser deposition (PLD). Afterwards, the deposited MgB_2_ films were treated with an ion-exchanger in an organic solvent to fabricate hydrogen boride films. Thin films wherein B-H bonds were introduced were successfully synthesised by using two types of ion-exchangers: proton exchange resin and formic acid. We also observed significant hydrogen release from these films under ultraviolet (UV) light irradiation.

## 2. Results and Discussions

**Figure 1:**
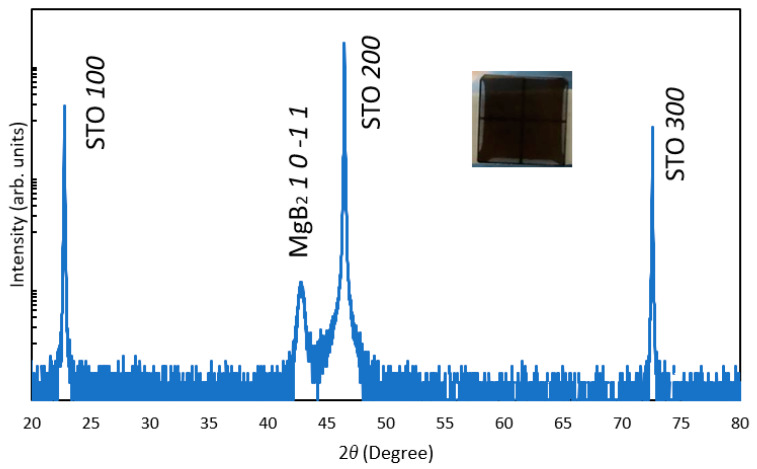
Out-of-plane XRD pattern of prepared MgB_2_ film. The inset image shows the appearance of the as-deposited MgB_2_ film on the STO substrate.

To fabricate the single-phase MgB_2_ film, a two-step PLD method was used as described in the experimental section. Figure 1 shows the out-of-plane XRD patterns of the MgB_2_ film deposited on the STO (100) substrate. As shown in the inset image, a black film was formed on the STO substrate. Only the *10-11* diffraction peak of the MgB_2_ film was detected, except for the peaks associated with the substrate, indicating its strong orientation. Such oriented growth of MgB_2_ is in agreement with previous reports [18,19,20]. Although a strong *10-11* peak appeared, our in-plane XRD analysis could not detect the diffraction peaks because the film thickness was insufficient for in-plane diffraction. Consecutively, we were unable to investigate the lattice matching relationship in the plane, and the film was described as a single-oriented thin film. The thickness of the MgB_2_ film varied between 50 nm and 270 nm (see Supplementary Information, Figure S1). When the film was prepared by a one-step PLD method (MgB_2_ deposition without Mg deposition), a peak of boron-rich magnesium boride (MgB*_x_*) was observed in addition to the MgB_2_ peak (Figure S2). The most likely reason for this is the lack of Mg in the one-step PLD method due to the high volatility of Mg. The expected candidates of the produced MgB*_x_* compound are listed in the Supplementary Information (Table S1). When a different crystalline substrate, such as sapphire, was used in a two-step PLD process, no diffraction peaks of MgB_2_ were observed (Figure S3). These results indicate that a dense and oriented MgB_2_ film can be deposited on the STO (100) substrate by a two-step PLD method.

**Figure 2:**
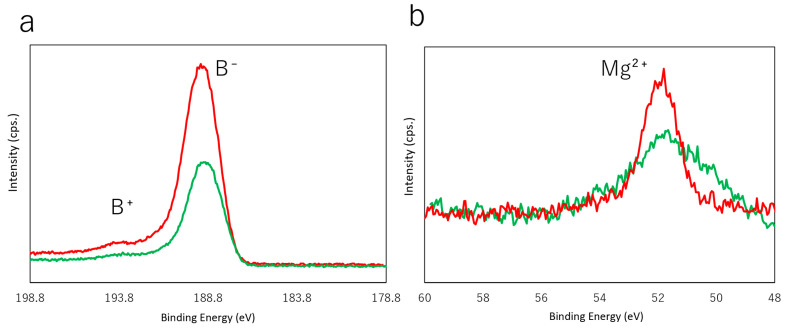
HAXPES spectra of B 1s (**a**) and Mg 2p (**b**) core-levels for the MgB_2_ film before (red) and after (green) ion-exchange treatment using resin.

Next, the oriented MgB_2_ film was treated with ion-exchange resin in acetonitrile. MgB_2_ is a layered compound in which positive magnesium ions with a valence number close to divalent are intercalated between negatively charged hexagonal boron layers, forming a honeycomb structure [21,22,23]. In the present study, an ion-exchange resin was ground in an agate mortar to increase the reaction area for efficient ion-exchange in a reaction time equivalent to that of powder sample synthesis. During the ion-exchange treatment, Mg^2+^ ions in MgB_2_ are changed with protons. Therefore, we expect that negatively charged boron states (B^−^) can remain even after the process, similar to previous powder sample experiments [2]. HAXPES analysis was performed to characterise the valence states of the boron and magnesium species in the film. Figure 2a shows the HAXPES spectra of the B 1s core-level before and after the ion-exchange treatment. As a result, the majority of the boron sites retained their negatively charged B^−^ state (peak at ~188.9 eV), even after the treatment. This indicates that oxidation does not occur during the ion-exchange, and impurities, such as boric acid, are not formed (B 1s of boric acid is known to appear at 193.2 eV [2]). As shown in Figure 2b, after the ion-exchange the Mg 2p core-level peak broadens, indicating that the surroundings of Mg in the thin film change significantly. X-ray diffraction data revealed a gradual decrease of the MgB_2_ *10-11* peaks after the treatment, suggesting that its crystallinity becomes worse after ion-exchange (see Supplementary Information, Figures S4 and S5).

**Figure 3:**
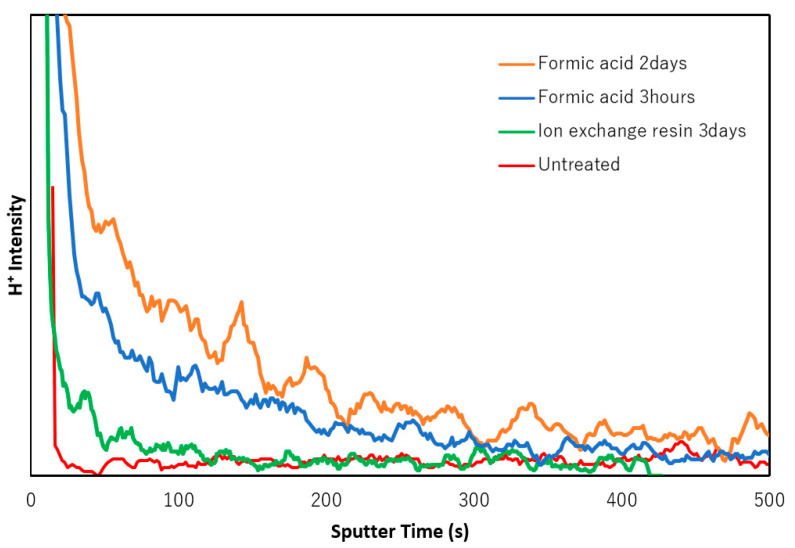
TOF-SIMS analysis of H^+^ species distribution in thin films: untreated thin film (red line), thin film treated by ion-exchange resin for 3 days (green line), thin film treated by formic acid for 3 h (blue line), and thin film treated by formic acid for 2 days (orange line).

Since photo-emission spectroscopy analysis is known to have low sensitivity to hydrogen species, TOF-SIMS analysis of the thin MgB_2_ films was performed. Figure 3 shows the TOF-SIMS depth profile of the ionised hydrogen species (H^+^) in the films. The red line demonstrates the H^+^ species profile of the MgB_2_ film. In this case, the observed H^+^ species on the surface originate from adsorbed water and/or hydrocarbon impurities. The ion-exchanged films exhibit a greater amount of hydrogen species than that of the untreated film. It can be assumed that the resin would not be an efficient reagent because of the limited exchange process at the solid-to-solid interface between MgB_2_ and the resin. Therefore, as an alternative ion-exchange method, liquid formic acid was used as a proton donor for more efficient ion-exchange. It is worth noting that the film treated with formic acid displayed a higher hydrogen content than the film treated with ion-exchange resin. The etching rate of the TOF-SIMS analysis was approximately 0.35–0.4 nm/s and was determined by the depth of the rastered area and sputtering time (Figure S6). Considering the etching rate, the 200 s sputtering time corresponds to 70–80 nm etching depth. The depth distribution of the hydrogen species varied depending on the method. In the formic acid-treated thin films, hydrogen species were introduced at a depth of less than 100 nm from the surface.

We also investigated the TOF-SIMS depth profiles of the boron and magnesium species (Figure S7). In the MgB_2_ film before the ion-exchange treatment, enrichment in boron species was observed near the surface (Figure S7a, red line) due to the volatilization of Mg in the outermost surface of the MgB_2_ thin film during the PLD process, as discussed above. After the ion-exchange process with the resin, both the boron and magnesium species’ contents decreased. The decrease in the abundance of the magnesium species is due to leaching via the ion-exchange with protons inhomogeneously, while the lower boron species content can be explained by the exfoliation of boron-rich layers from the thin film. The decrease in the boron species signal was more significant compared to that of the magnesium species. The integrated values of the TOF-SIMS signal intensities from 0 to 20 s (corresponding to the 7–8 nm layer on the top surface) before and after the ion-exchange treatments were calculated and compared. Then, the relative values after the resin ion-exchange with respect to the values before the ion-exchange were 0.51 and 0.71 for the boron species and magnesium species, respectively.

TOF-SIMS analysis of the boron and magnesium species were also performed for thin films treated with formic acid (Figure S8). Similar to the resin treatment, the content of both boron and magnesium species was reduced after the formic acid treatment. In this case, the decrease in the magnesium signal was more significant than that of the boron species. These results indicate that a long-term treatment with formic acid can induce a proton ion-exchange process rather than an exfoliation of the boron layers in the MgB_2_ film. We also evaluated the TOF-SIMS signal of the carbon species after the sample was treated with formic acid (Figure S9). The signal intensities of the carbon species were very low, even after the formic acid treatment. As shown in Figure 3, the hydrogen species signal was stronger and detectable at a greater depth below the surface compared to the carbon species signal (Figure S9). These results indicate that the dissociated protons from formic acid were successfully introduced into the MgB_2_ film during the ion-exchange process.

Next, FT-IR measurements were conducted in an attempt to confirm the formation of B-H and/or B-H-B bonds in the ion-exchanged film as an additional spectroscopic evidence for the ion exchange of Mg^2+^ by protons. In spite of using the highly sensitive ATR method, no signals characteristic of these bonds were detected, presumably because the film was too thin. Instead, the FT-IR spectrum of MgB_2_ powder was investigated (Figure S10), commercially available from Sigma-Aldrich and handled in the same manner as the thin films. Notably, after the ion-exchange treatment in formic acid, a peak appeared around 2500 cm^−^^1^. According to a previous report, this signal corresponds to the terminal B-H stretching mode in hydrogen boride sheets [4]. Since we followed an identical ion-exchange process for both the powder sample and the thin film, we assume that a similar B-H bond was formed in the film sample, as the existence of hydrogen species was also confirmed in the film by TOF-SIMS measurements.

**Figure 4:**
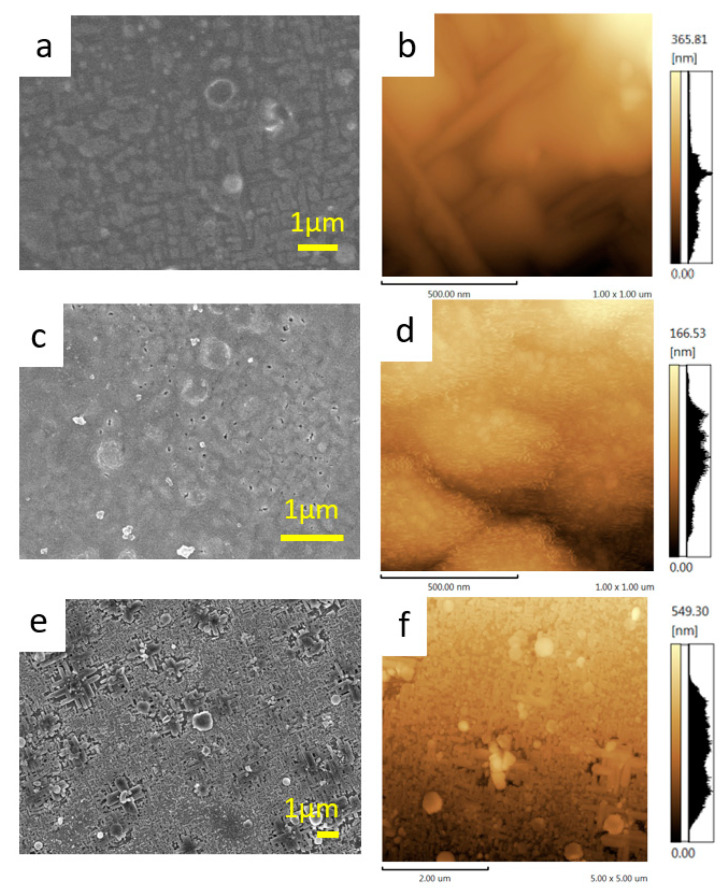
SEM (**a**,**c**,**e**) and AFM (**b**,**d**,**f**) images of thin films. As-deposited MgB_2_ film without ion-exchange treatment (**a**,**b**), ion-exchanged film treated with resin for 3 days (**c,d**), and ion-exchanged film treated with formic acid for 2 days (**e**,**f**).

We also compared the surface morphologies of the as-deposited and ion-exchanged films by SEM and AFM, as shown in Figure 4. The SEM (Figure 4a) and AFM (Figure 4b) images of the as-deposited MgB_2_ thin film (before the ion-exchange) reveal cross-shaped rectangular patterns. This implies that the MgB_2_ film has an epitaxial relationship with the STO substrate, although we could not confirm this by in-plane XRD. The peak to valley (PTV) height difference in the AFM image was 50–100 nm (Figure 4b, Figure S11a). In contrast, the film treated with the ion-exchange resin exhibited a smoother surface compared to the as-deposited MgB_2_ film, with a PTV height difference of less than 50 nm (Figure 4c,d and Figure S11b). This is likely because of the physical contact (mechanical abrasion) between the MgB_2_ thin film and the resin during the ion-exchange. The thickness of the film after the ion-exchange treatment was less than 200 nm (Figure S12). This might be one of the reasons for the lower H content and its narrower depth distribution observed in the TOF-SIMS profile. On the other hand, the film treated with formic acid for 2 days exhibited more pronounced crisscross patterns on the surface and a larger PTV height difference (50–200 nm), as shown in Figure 4e,f and Figure S11c. This is because the ion-exchange and exfoliation were more efficient when using formic acid, as compared to the use of ion-exchange resin. These results reveal that both the hydrogen content and the surface morphology of the thin films can vary significantly depending on the type of ion-exchange treatment. This also suggests that, by choosing an appropriate ion-exchange method and conditions, the morphology and hydrogen content of the films can be controlled for various device applications.

**Figure 5:**
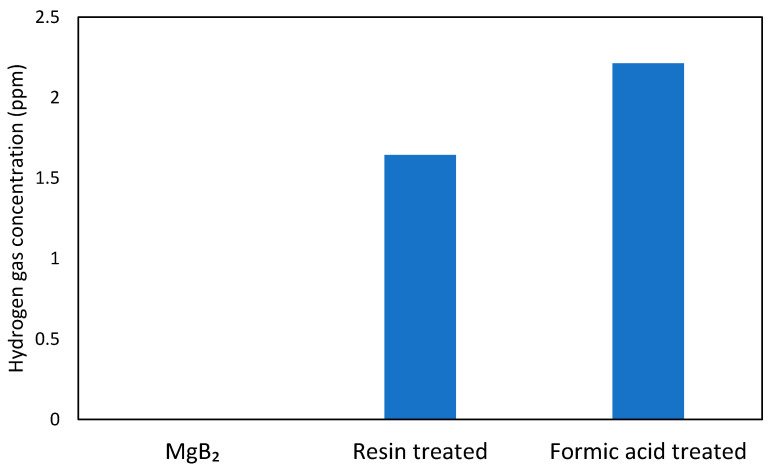
Hydrogen release from as-deposited MgB_2_ and ion-exchanged thin films under UV irradiation.

Previous experiments on powder systems have demonstrated that UV irradiation of the B-H bonds in hydrogen boride sheets can lead to hydrogen gas release [7]. In this study, the hydrogen release was induced by photoirradiation, which causes the electron transition from the σ-bonding state (valence band) to the anti-bonding state of boron and hydrogen orbitals (conduction band) with a gap of 3.8 eV and causes the self-reduction of protons together with oxidation of boron in HB, even under mild ambient conditions. We anticipated that a similar mechanism might lead to hydrogen emission from the ion-exchanged thin films upon UV irradiation. As shown in Figure 5, hydrogen molecules were detected from both types of ion-exchanged films upon UV light irradiation, while the as-deposited MgB_2_ did not release hydrogen gas. The amount of hydrogen released from the formic acid treated film was larger than that released from the resin-treated film. These results agree well with the TOF-SIMS data, which showed a higher hydrogen species content in the film treated with formic acid compared to the other one. That strongly implies that B-H bonds were formed in the ion-exchanged film, as suggested by the FT-IR analysis, and hydrogen molecules can be liberated by an electron transition from the bonding state to the anti-bonding state of hydrogen and boron orbitals under UV light irradiation [7].

**Figure 6:**
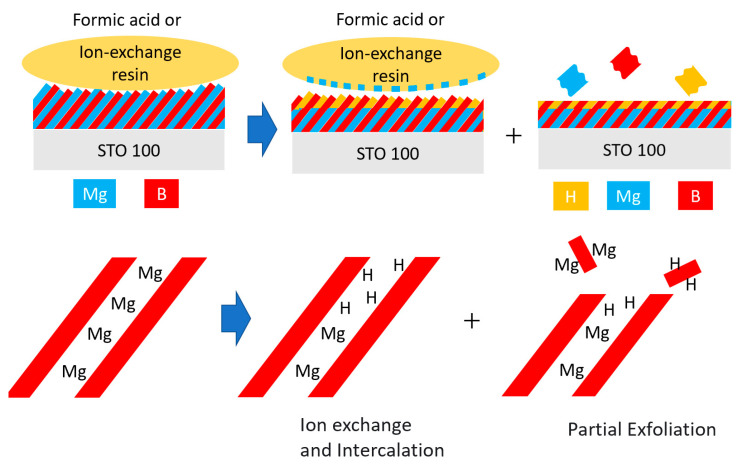
Proposed reaction scheme of the ion-exchange mechanism.

Based on the experimental data, we can propose a reaction scheme for the ion-exchange process in MgB_2_ films, as shown in Figure 6. The oriented MgB_2_ thin film reacts with ion-exchange resin or formic acid (left panel) causing H^+^ to intercalate inside the film and Mg^2+^ ions to be released from the film in the acetonitrile solution (middle panel), similar to a layered metal oxide case [24]. In addition to the ion-exchange, a partial exfoliation of boron layers occurred at the outermost surface, leaving H^+^ inside the thin film to form B-H bonds (right panel). In terms of the chemical composition, the ion-exchanged film is depicted as an inhomogeneous hybrid material with a top layer mainly consisting of HB and a bottom layer mainly consisting of MgB_2_.

## 3. Materials and Methods

### 3.1. Materials and Equipment

SrTiO_3_ (100) single-crystal substrates were purchased from SHINKOSHA CO., LTD. Mg powder (212–600 μm, 99.9%, Wako, Osaka, Japan) and MgB_2_ powder (99%; Sigma-Aldrich, St. Louis, MO, USA) were purchased to use MgB_2_ thin film deposition. Acetonitrile (99.5% (JIS Special grade), FUJIFILM Wako Pure Chemical Industries Ltd., Osaka, Japan), a cation-exchange resin (15JS-HG⋅DRY, Organo Corp., Tokyo, Japan) and formic acid (Kanto Chemical Co., Inc., Tokyo, Japan) were purchased for ion-exchange treatment.

A pulsed laser ablation device (PLFD-221-1R, Freedom Ltd., Kawasaki, Japan) was used for PLD. X-ray diffractometer (Smartlab, Rigaku Corporation, Tokyo, Japan), laser microscope (LEXT OLS5100, Olympus Corporation, Tokyo, Japan), TOF-SIMS 5-100-AD (ION-TOF GmbH, Germany), FT/IR-6100 (JASCO, Co., Ltd., Tokyo, Japan), atomic force microscope (SPM-9700, Shimadzu Corp., Kyoto, Japan) and scanning electron microscope (JEM-2010F, JEOL, Ltd., Tokyo, Japan) were used for characterization of prepared films.

### 3.2. Preparation of MgB_2_ Thin Films

The MgB_2_ thin films were deposited on SrTiO_3_ (100) single-crystal substrates by PLD using the fourth harmonic wavelength (266 nm) of a neodymium: yttrium aluminium garnet (Nd: YAG) laser. The substrate size was 1.0 × 1.0 cm^2^. The thin film deposition was performed under argon partial pressure of 1.0 mTorr and substrate temperature of 700 °C. A high-purity single-phase MgB_2_ film was deposited via a two-step deposition using subsequently two targets: a metallic magnesium (Mg) target pellet for 10 min and an MgB_2_ pellet for 60 min for the same substrate. The former process was necessary to compensate for the Mg supply in the MgB_2_ film because of the high volatility of Mg. The Mg pellet was prepared by uniaxial pressing using Mg powder, while the MgB_2_ pellet was prepared using commercial powder.

### 3.3. Ion-Exchange Treatment of MgB_2_ Thin Films

Two different types of ion-exchange method were employed to treat the deposited MgB_2_ thin films: the first method involved a 3-day reaction with a cation-exchange resin (400 mg) that was ground in an agate mortar in acetonitrile while referencing the previous report [2]. As an alternative ion-exchange method, a solution of 3.6 mL of acetonitrile and 0.4 mL of formic acid as a proton donor was prepared according to the report by Kawamura et al. [25], and MgB_2_ thin films were immersed into the solution for either 3 h or 2 d. Both treatments were conducted at room temperature and atmospheric pressure in a glovebox (<1 ppm O_2_) to prevent air exposure. The ion-exchanged film samples were washed with acetonitrile and dried under a nitrogen atmosphere.

### 3.4. Characterization of Prepared Films

X-ray diffraction (XRD) measurements were performed using a Rigaku Smartlab X-ray diffractometer (45 kV, 200 mA, radiation source: Cu(Kα_1_)). The film thickness was measured using a laser microscope. We evaluated the height difference between the MgB_2_ thin film and the bare STO substrate near the edge of the thin film.

Hard X-ray photoemission spectroscopy (HAXPES) measurements were performed at BL15XU of SPring-8 (Super Photon Ring 8 GeV, Hyōgo Prefecture, Japan). The excitation photon energy and total energy resolution were set to 5.95 keV and 240 meV, respectively. The measurements were taken at room temperature and the pressure of the analysis chamber of HAXPES was 1.1 × 10^−^^7^ Pa. Details of HAXPES experiments at BL15XU were described elsewhere [26].

Time-of-flight secondary ion mass spectroscopy (TOF-SIMS) depth profile analyses were performed using a TOF-SIMS 5-100-AD. The analysis parameters were as follows: primary ion source, Bi_3_^++^; energy, 60 keV; area, 100 × 100 μm^2^. The sputtering parameters were as follows: sputtering ion source, O_2_; energy, 2 keV; area, 300 × 300 μm^2^. To investigate the relationship between the actual depth and the sputtering time of the TOF-SIMS, the depth of the rastered area by ion beam was measured using a laser microscope and the sputtering rate was estimated.

Fourier-transform infrared spectroscopy (FT-IR) spectra were recorded using an FT/IR-6100 (MCT detector). The MgB_2_ film was too thin for the detection of the FT-IR signals of B-H and/or B-H-B bonds. Thus, an additional FT-IR analysis was conducted using ion-exchanged MgB_2_ powder by an attenuated total reflection (ATR) method using a diamond holder. For FT-IR analysis, MgB_2_ powder was treated in the same manner as the MgB_2_ film. Atomic force microscopy (AFM) images were obtained in dynamic imaging mode using an SPM-9700 with a silicon cantilever. Scanning electron microscope (SEM) images were obtained using a JEM-2010F (6.0 kV).

### 3.5. Photoinduced Hydrogen Gas Release

A film sample was placed in a quartz cell (3.5 mL with septum screw cap) under a nitrogen atmosphere without any solvent. Hydrogen production in the quartz cell under near-UV light irradiation was evaluated by gas chromatography (Tracera-GC-2010 Plus with a BID detector, Shimadzu, Co., Ltd., Japan). A film sample was placed under dark conditions for 1 h, followed by UV irradiation for 2 h. Hydrogen generation was determined by the difference in hydrogen amounts before and after the UV irradiation. UV irradiation was performed using a mercury-xenon (Hg-Xe) lamp with a 340 nm band-pass filter, similar to previous reports [7]. As a control experiment, the MgB_2_ film without an ion-exchange treatment was also evaluated in the same manner as the ion-exchanged films.

## 4. Conclusions

We established a fabrication process for protonated boride films by using a facile ion-exchange under ambient temperature and atmospheric pressure. This is the first report of HB thin film fabrication on a substrate. The oriented MgB_2_ thin films were prepared by a two-step PLD method and protons were successfully introduced into the films by treatment with either ion-exchange resin or formic acid in an organic solvent. Depending on the treatment conditions, the films with different characteristics such as proton content and surface structure have been obtained. The irradiation treatment on thin film with near-UV light produces hydrogen gas, similar to the powder HB samples. The present study is the first to demonstrate light-induced hydrogen release from a protonated boride thin film. This study will provide a method to fabricate thin films with B-H bonds, which will be useful for further studies or device applications.

## Supplementary Materials

The followings are available online: Table S1: XRD peaks data, Figures S1, S6 and S12: microscope images, Figures S2–S5: XRD patterns, Figures S7–S9: SIMS depth profiles, Figure S10: FT-IR spectra, Figure S11: AFM images.

## Sample Availability

Samples of the compounds are not available from the authors.
