# Supplementary material for: Fabrication of Hydrogen Boride Thin Film by Ion Exchange in MgB2
Source: Molecules. 2021 Oct 14;26(20):6212. doi: 10.3390/molecules26206212 (PMC8540303; doi:10.3390/molecules26206212)
Supplement: Supplementary file 1 [file molecules-26-06212-s001.zip › molecules-1397143-supplementary.pdf]

Supplementary Information

Fabrication of Hydrogen Boride Thin Film by Ion Exchange in MgB_2

T. Hirabayashi [1,2,†], S. Yasuhara [1,†], S. Shoji [1,3]A. Yamaguchi [1], H. Abe [4], S. Ueda [5,6,7], H. Zhu[2], T. Kondo [8,9,*] and M. Miyauchi[1,*]

[1] Department of Materials Science and Engineering, School of Materials and Chemical Technology, Tokyo Institute of Technology, 2-12-1, Ookayama, Meguro-ku, Tokyo 152-8552, Japan.

[2] Department of Mechanical Engineering, School of Materials Science and Engineering, Tsinghua University, Beijing 100084, China.

[3] Department of Materials Science & Engineering, Cornell University, Ithaca, NY 14853, USA.

[4] Center for Green Research on Energy and Environmental Materials, National Institute for Materials Science, 1-1, Namiki, Tsukuba, Ibaraki 305-0044, Japan.

[5] Synchrotron X-ray Station at SPring-8, National Institute for Materials Science, 1-1-1 Kouto, Sayo, Hyogo 679-5148, Japan.

[6] Research Center for Advanced Measurement and Characterization, National Institute for Materials Science, 1-2-1 Sengen, Tsukuba, Ibaraki 305-0047, Japan.

[7] Research Center for Functional Materials, National Institute for Materials Science, 1-1 Namiki, Tsukuba, Ibaraki 305-0044, Japan.

[8] Department of Materials Science and Tsukuba Research Center for Energy Materials Science, Faculty of Pure and Applied Sciences, University of Tsukuba, 1-1-1, Tennodai, Tsukuba, Ibaraki 305-8573, Japan.

[9] Materials Research Center for Element Strategy, Tokyo Institute of Technology, 4259 Nagatsuta-cho, Midori-ku, Yokohama, Kanagawa 226-8503, Japan.

* Correspondence: takahiro@ims.tsukuba.ac.jp, mmiyauchi@ceram.titech.ac.jp

[†] The 1st and 2nd authors equally contributed to this work.

Table S1 Expected candidates of B-rich magnesium boride compounds (MgB_x) produced due to insufficient Mg supply and their XRD peaks

	2θ(degree)	d(Å)	(h k l)
MgB_3	42.07	2.148	(1 1 2)
	42.61	2.122	(0 0 3)
	44.01	2.058	(2 0 0)
MgB_4	41.89	2.157	(0 1 3)
MgB_7	41.32	2.185	(2 3 1)
	42.29	2.137	(0 3 3)
	44.71	2.027	(0 5 1)

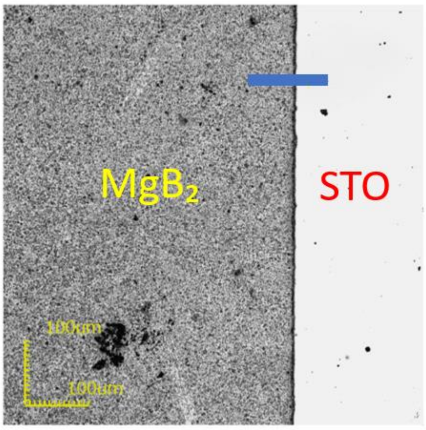

	No.	Result	Width[μm]	Height[μm]	Length[μm]	Angle[°]	File name
✓	1		68.415	0.266	68.415	0.223	210428_1626
Count			1	1	1	1	1
Average			68.415	0.266	68.415	0.223	
Min.			68.415	0.266	68.415	0.223	
Max.			68.415	0.266	68.415	0.223	
Range			0.000	0.000	0.000	0.000	
σ			0.000	0.000	0.000	0.000	
3σ			0.000	0.000	0.000	0.000	
Tolerance			Off	Off	Off	Off	Off
Upper tolerance			0	0	0	0	
Standard			0	0	0	0	
Lower tolerance			0	0	0	0	

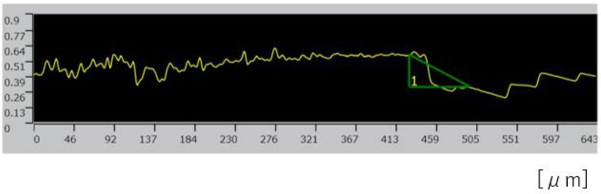

Figure S1 Microscope image at the edge of the MgB_2 film (left photo), and its depth profile analyzed by laser microscope (right image). Blue line in the photo represents the line for depth profile shown in the right image.

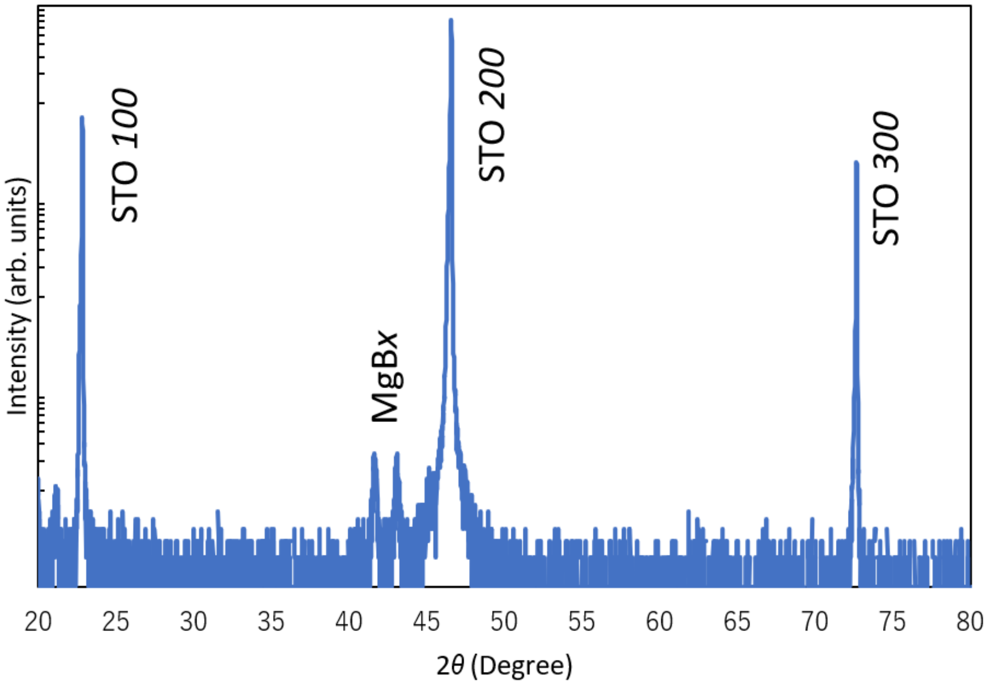

Figure S2 Out-of-plane XRD pattern of the thin film fabricated via the one step PLD method

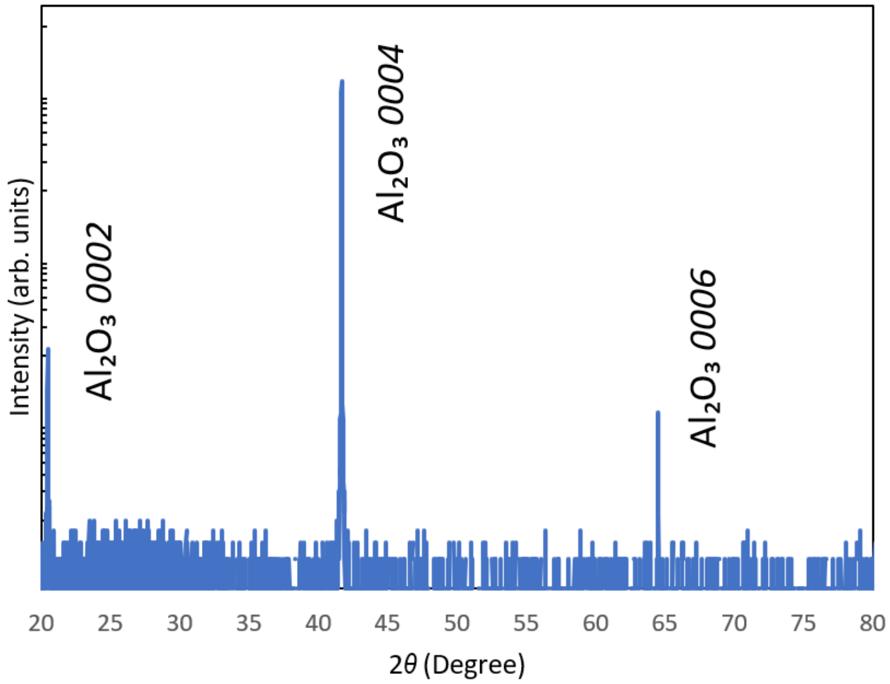

Figure S3 Out-of-plane XRD pattern of the film prepared using a sapphire substrate by the two-step PLD method.

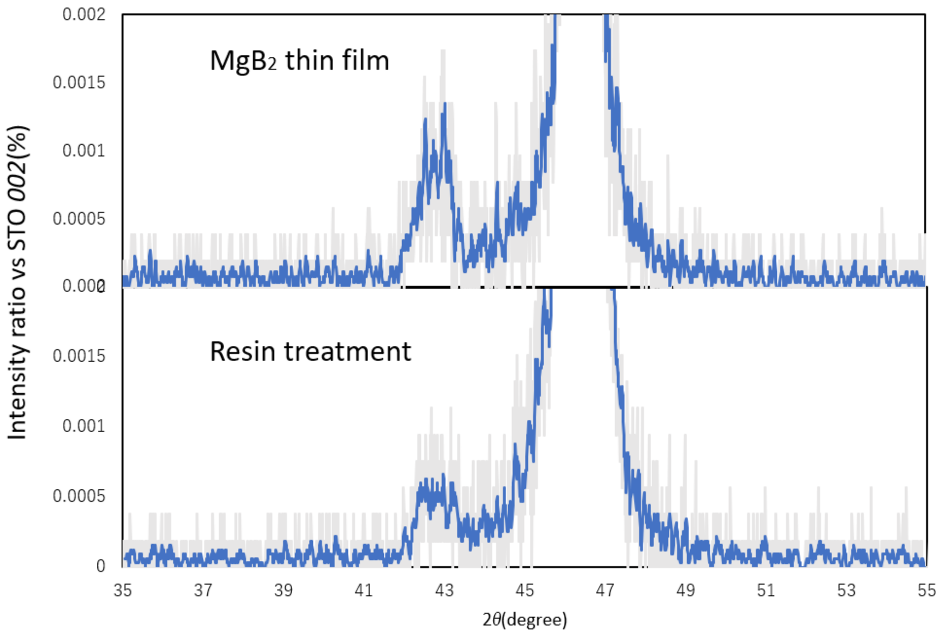

Figure S4 XRD patterns of as-deposited MgB_2 film (upper) and the film treated with ion-exchange resin for 3 days (lower). The peak intensities in XRD patterns were normalized as that of the (200) face of a $SrTiO_3$ (STO) substrate to be 100 %. Gray lines are raw data. Blue lines represent moving average of raw data.

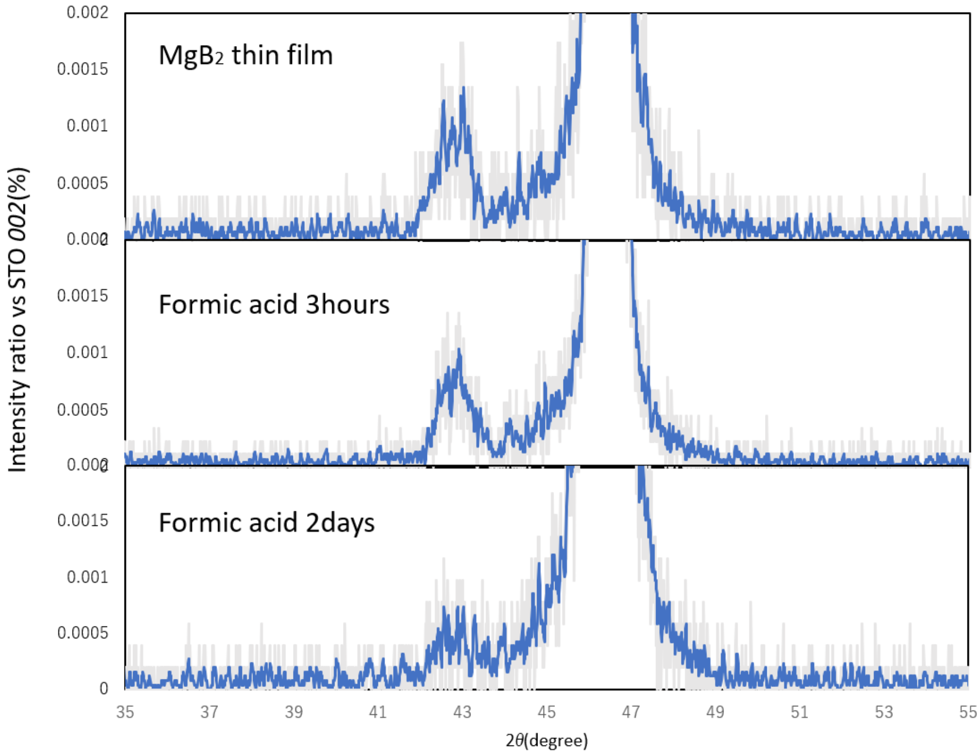

Figure S5 XRD patterns of as-deposited MgB_2 film and the film treated with formic acid. The peak intensities in XRD patterns were normalized as that of the (200) face of a $SrTiO_3$ (STO) substrate to be 100 %. Gray lines are raw data. Blue lines represent moving average of raw data.

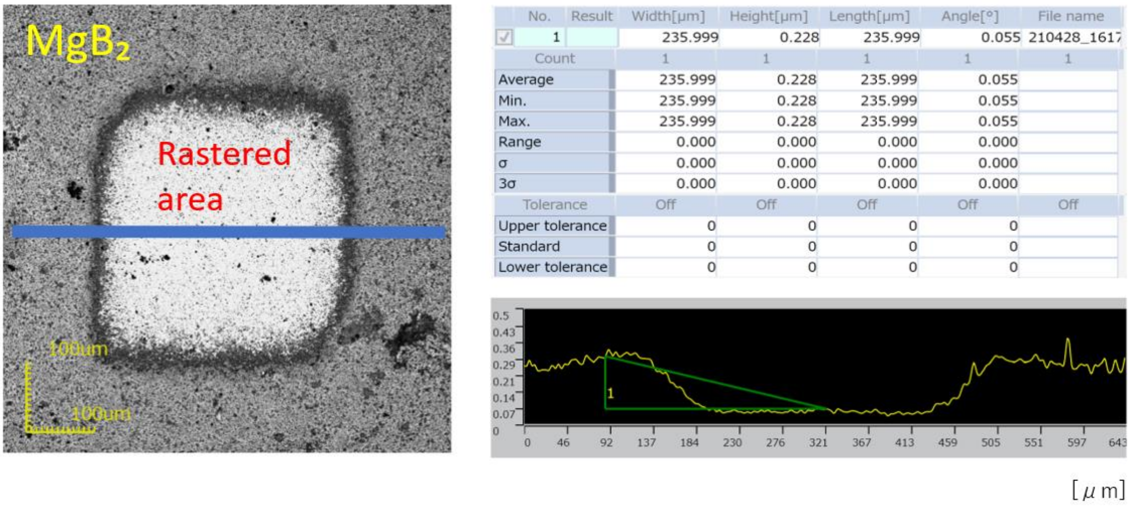

Figure S6 The TOF-SIMS measurement rastered area image and depth profile observed by laser microscope. Blue line in the photo represents the line for depth profile shown in the right image.

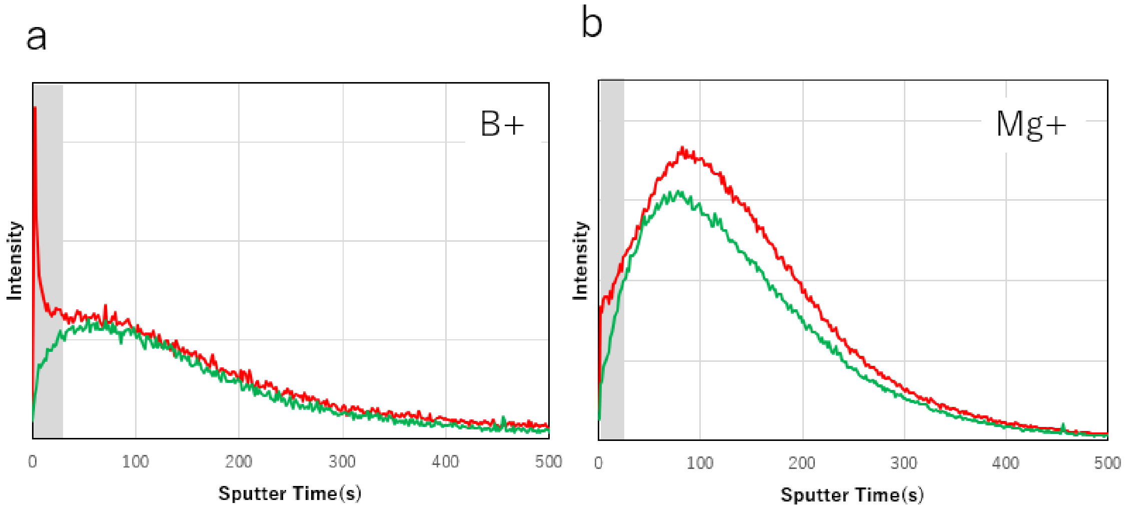

Figure S7 TOF-SIMS analyses for B+ (a) and Mg+ (b) species in thin films. Red lines represent the profiles of the MgB_2 film before ion-exchange treatment, while green lines represent the those of ion-exchanged film using ion-exchange resin for 3 days.

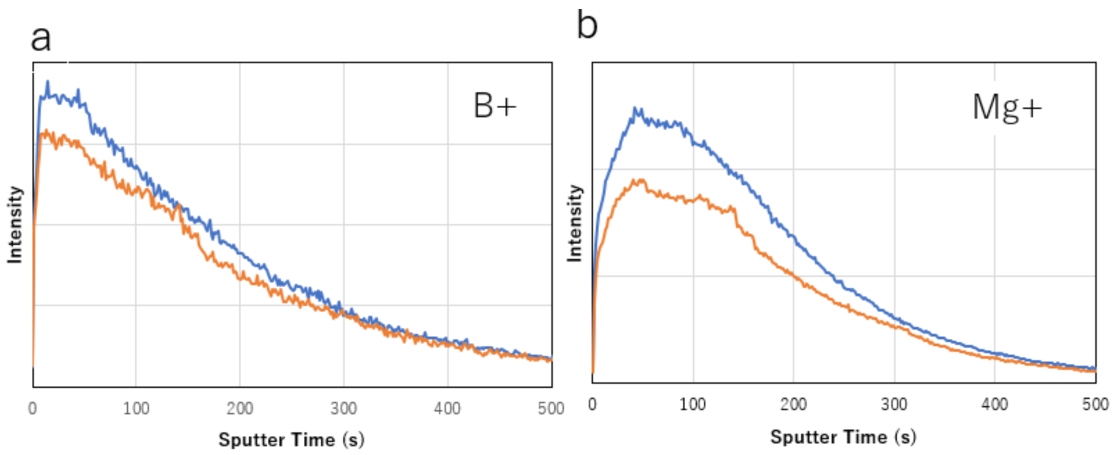

Figure S8 TOF-SIMS analyses for B+ (a) and Mg+ (b) species in thin films. Blue lines represent the profiles of the thin film after 3 h treatment in formic acid, while orange lines represent the those after 2 days in formic acid.

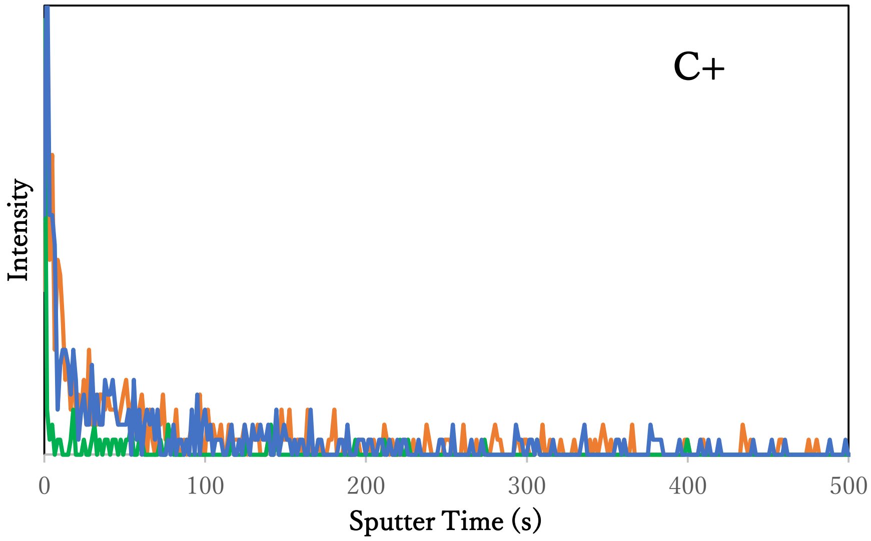

Figure S9 TOF-SIMS analyses for C+. The green line represents the profiles of the film after 3 days treatment with resin, and blue and orange lines were the results of 3 h and 2 days with formic acid.

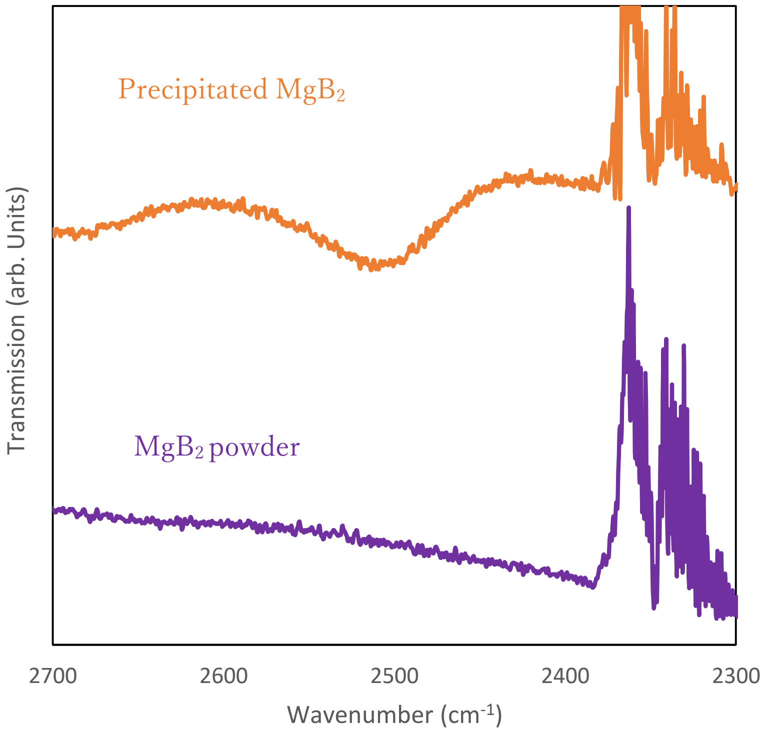

Figure S10 FT-IR spectra of the commercial MgB_2 powder (purple line) and that treated with formic acid (orange line).

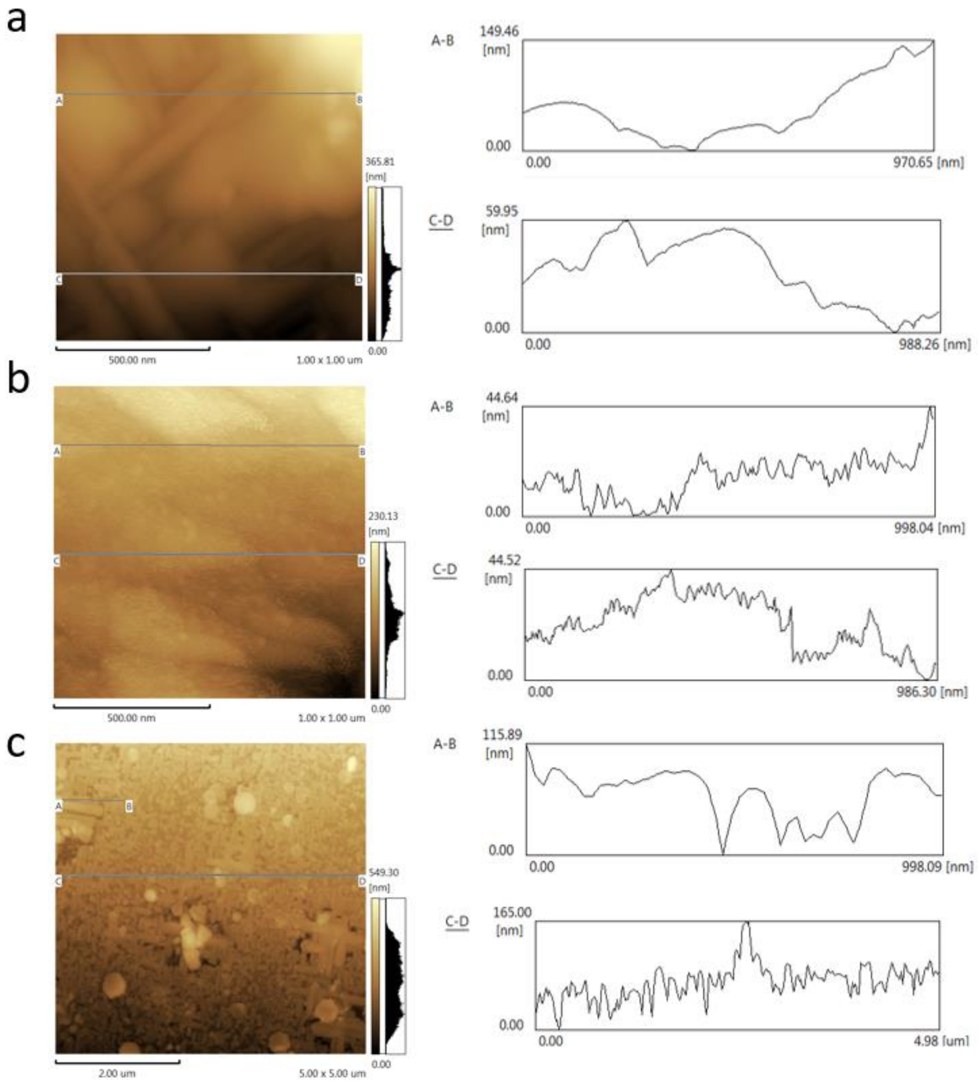

Figure S11 AFM images of thin films and their line profiles (a: MgB_2 thin film, b: ion-exchanged film by resin, c: ion-exchanged film by formic acid).

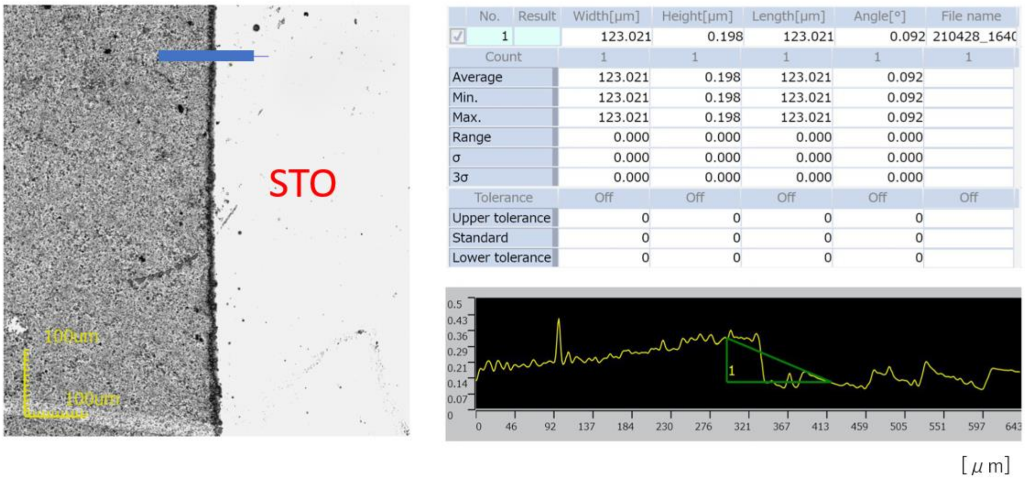

No.	Result	Width[µm]	Height[µm]	Length[µm]	Angle[°]	File name
1		123.021	0.198	123.021	0.092	210428_1640
Count		1	1	1	1	1
Average		123.021	0.198	123.021	0.092	
Min.		123.021	0.198	123.021	0.092	
Max.		123.021	0.198	123.021	0.092	
Range		0.000	0.000	0.000	0.000	
σ		0.000	0.000	0.000	0.000	
3σ		0.000	0.000	0.000	0.000	
Tolerance		Off	Off	Off	Off	Off
Upper tolerance		0	0	0	0	
Standard		0	0	0	0	
Lower tolerance		0	0	0	0	

Figure S12 Microscope image at the edge of the film treated by ion-exchange resin (left photo), and its depth profile analyzed by laser microscope (right image). Blue line in the photo represents the line for depth profile shown in the right image.
